# What do docking and QSAR tell us about the design of HIV-1 reverse transcriptase nonnucleoside inhibitors?

**DOI:** 10.1007/s00894-017-3489-3

**Published:** 2017-10-19

**Authors:** Agata Paneth, Wojciech Płonka, Piotr Paneth

**Affiliations:** 1 Institute of Applied Radiation Chemistry, Lodz University of Technology, Żeromskiego 116, 90-924 Łódź, Poland; 2 Faculty of Pharmacy, Medical University of Lublin, Chodźki 4a, 20-093 Lublin, Poland; 3 FQS-Fujitsu Poland, Parkowa 11, 33-332 Kraków, Poland

**Keywords:** HIV-1 reverse transcriptase, Docking, QSAR

## Abstract

Despite vigorous studies, effective nonnucleoside inhibitors of HIV-1 reverse transcriptase (NNRTIs) are still in demand, not only due to toxicity and detrimental side effects of currently used drugs but also because of the emergence of multidrug-resistant viral strains. In this contribution, we present results of docking of 47 inhibitors to 107 allosteric centers of HIV-1 reverse transcriptase. Based on the average binding scores, we have constructed QSAR equations to elucidate directions of further developments in the inhibitor design that come from this structural data.

**Electronic supplementary material:**

The online version of this article (10.1007/s00894-017-3489-3) contains supplementary material, which is available to authorized users.

## Introduction

According to the latest estimates from The Joint United Nations Programme on HIV and AIDS, there were 36.7 million people living with HIV in 2015, up from 33.3 million in 2010. Among them, 1.1 million died from AIDS-related illnesses [1, 2]. The currently licensed antiviral medications include drugs falling into five main classes targeting different steps in the HIV life cycle: reverse transcription (nucleoside/nucleotide and non-nucleoside reverse transcriptase inhibitors), assembly, budding, and maturation (protease inhibitors), viral entry (fusion inhibitors and co-receptor antagonists), and integration (integrase inhibitors). Standard first-line antiretroviral therapy (ART) for adults consists of three reverse transcriptase inhibitors (two nucleosides plus nonnucleoside) or two nucleoside reverse transcriptase inhibitors in combination with an integrase inhibitor [3, 4]. Although ART has its limitations, it has proved largely effective, particularly in the early stages of the disease, reducing the mortality from AIDS and turning the disease from lethal to chronic [5]. Because of the rapid emergence of multidrug-resistant viral strains, toxicity, and detrimental side effects caused by long-term drug treatment [6], however, the discovery of new antiviral agents, in particular those targeting HIV-1 reverse transcriptase, is a research priority.

HIV-1 reverse transcriptase (RT) is an asymmetric heterodimeric enzyme composed of a catalytically active 66-kDa subunit with three domains: polymerase (residues 1–319), connection (residues 320–440) and RNase H (residues 441–560), and a catalytically inactive 51-kDa subunit containing non-functional polymerase and connection domains [7]. Nearly half of approved antiretroviral drugs target the polymerase active site of RT or the allosteric cavity located 10 Å away [8]. Five of them are non-nucleoside RT inhibitors that bind to the allosteric site, called the non-nucleoside inhibitor binding pocket (NNIBP), in a noncompetitive manner, resulting in limitation of conformational flexibility required for efficient DNA synthesis by RT [9]. Nevirapine (NVP), delavirdine (DLV), and efavirenz (EFV) are first-generation non-nucleoside reverse transcriptase inhibitors (NNRTIs) with potent anti-HIV-1 activity, but their adverse effects, particularly those affecting the central nervous system and hepatotoxicity, poor resistance profile, and low genetic barriers for viral resistance, especially single mutants K103N, Y181C, and double mutant K103N/Y181C, limited their clinical application [10]. The second-generation NNRTIs etravirine (ETR) and rilpivirine (RPV) have been approved by the Food and Drug Administration and European Union in 2008 and 2011, respectively. Both display a broad spectrum of activity against clinically relevant HIV*-*1 mutant and wild-type (wt) HIV-1 strains at low nanomolar concentrations. However, etravirine must be given twice daily and is not recommended for initial treatment of HIV infection because of insufficient data in treatment-naive individuals whilst rilpivirine has suboptimal efficacy in patients with viral loads greater than 100,000 copies/ml or CD4 cell counts below 200 copies/ml at baseline because of higher virologic failure rate. Moreover, their use in some settings is limited by hypersensitivity reactions or other adverse effects. In addition, although these drugs have higher genetic barriers to resistance than first-generation NNRTIs, drug resistance still emerges, and K103N and E138K are the most common mutations selected by ETV and RPV. Importantly, when resistance to RPV is selected after virologic failure, cross-resistance to all nonnucleoside reverse transcriptase inhibitors is commonly observed [11–21]. Therefore, there is a considerable need for new drugs with anti-HIV-1 reverse transcriptase activity. To address this problem, in this contribution we present results of docking of 47 inhibitors bound to 107 allosteric centers of HIV-1 reverse transcriptase, i.e., all data available in the Protein Data Bank. Based on the average binding scores, we have constructed QSAR equations to elucidate directions of further developments in the inhibitor design that come from this structural data.

## Methods

All structures of HIV-1 reverse transcriptase with ligand bound in the allosteric site available in the Protein Data Bank (www.rcsb.org) [22] have been used. These include 72 structures of the wild-type enzyme: 1bqm, 1c0t, 1dtq, 1dtt, 1fk9, 1hpu, 1hpz, 1ikw, 1rt2, 1rt4, 1s6p, 1s9e, 1suq, 1sv5, 1tkt, 1tkx, 1tkz, 1tl1, 1tl3, 1vrt, 1vru, 2be2, 2hnd, 2jle, 2opp, 2rf2, 2rki, 2vg5, 2vg6, 2vg7, 2wom, 2won, 2ykm, 2ykn, 2yng, 2ynh, 2yni, 2zd1, 3dle,3dlg, 3drp, 3hvt, 3irx, 3is9, 3lak, 3lal, 3lam, 3lan, 3lp0, 3lp1, 3m8p, 3m8q, 3mec, 3mee, 3qip, 3qlh, 3qo9, 3v81, 3v81, 4b3q, 4g1q, 4i2p, 4i2q, 4i7f, 4icl, 4ko0, 4puo, 4pwd, 4q0b, 5cym, 5cyq, and 5 k14 out of which three structures (4puo, 4pwd, and 4q0b) are tetramers with slightly different allosteric site architecture. For these three structures, binding of ligands to both sites has been studied. Furthermore, 32 structures of mutated enzyme: 1bqn, 1fko, 1fkp, 1ikv, 1jkh, 1jla, 1jlc, 1jlg, 1lw0, 1lwc, 1lwe, 1lwf, 1s1t, 1s1u, 1s1v, 1s1w, 1s1x, 2hny, 2ic3 2opq. 2opr, 2ops, 2ynf, 2ze2, 3bgr, 3 dm2, 3dmj, 3dok, 3dol, 3med, 3meg, and 5fdl were studied bearing 12 types of mutations with dominating mutations being K103N followed by Y181C. The set included six double mutations and one triple mutation.

In the above 107 structures, 48 ligands are present. They represent a number of different classes of chemicals. By far the most frequently studied (25 entries) ligand is NVP. Codes and structures of the remaining ligands are collected in Table 1. Out of compounds currently in clinical use, only DLV is not present in the set, as it was crystallized in the pocket of HIV-2 reverse transcriptase, which differs structurally from the allosteric pocket of HIV-1 enzyme [23]. Furthermore, we have excluded from the studies QO9 ligand, as it contains silicon atoms for which there is no parametrization. These ligands have been used in docking and subsequently in QSAR analysis.

**Table 1.**
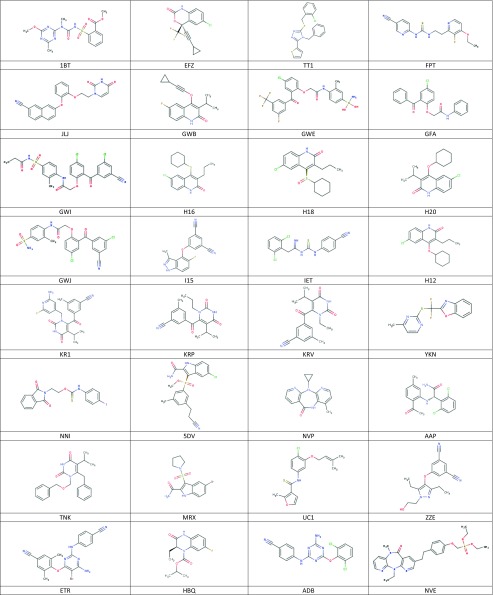

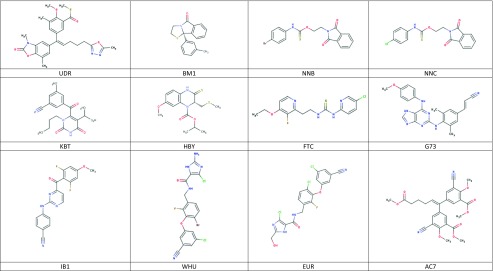
Structures of ligands used in docking and QSAR

Docking was performed using the FlexX program [24, 25], as implemented in the LeadIT software package [26]. The receptor was prepared using graphical interface of the package. Both protein chains were selected, and the binding site was defined to include residues within a 6.5-Å radius around the native ligand. The library of ligands was imported from the .mol2 file. Protonation states corresponding to aqueous solution were used. Soft docking (allowing for volume overlap up to 100 Å^3^) was performed. For ligand base placement, the default hybrid Enthalpy and Entropy strategy was used. The Clash Factor was set to 0.6. Other parameters were kept at default. Scores of the top-ranked poses of each ligand were averaged for a given enzyme structure.

QSAR analysis was performed in two different modes. In both cases, the training set included 47 structures; the end-point was the average docking score obtained from FlexX calculations after exclusion of results for QO9, which as the only one yielded positive values of the score function. The size of the data set was too small to allow splitting it into training and validation sets, so the internal leave-one-out cross-validation approach was chosen instead.

In the first approach, based on classical descriptors, several models were created using the *Complete Topological QSAR* method and regression equation, created by feature selection with Enhanced Replacement Method [ERM] as implemented in SCIGRESS Suite software [27]. In the second approach, QSAR [28] was based on molecular fragments contribution. The common substructures were extracted from a training set, yielding a set of 96 substructure-count descriptors by means of an algorithm based on RECAP using ADMEWORKS ModelBuilder [29, 30]. The substructures, present in less than three of training structures, were removed by means of zero-test with a threshold of 6%, leaving 39 substructure count descriptors. Particle Swarm Optimization algorithm [31] was employed for feature selection with a target of selecting 15 descriptors. After approximately 7000 iterations of 10,000 model population, the process was manually interrupted. Eighteen of the most often used descriptors were selected. The final model was created using leaps-and-bounds multiple linear regression model, a variation of backward stepwise regression.

## Results and discussion

**Fig. 1:**
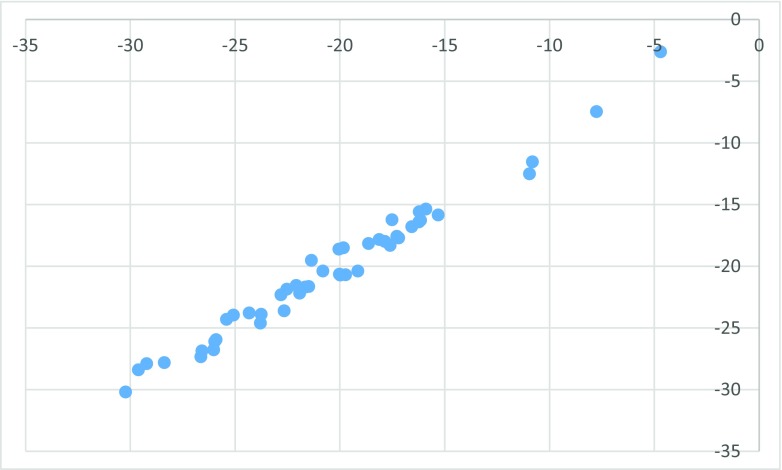
Averaged FlexX score of binding to mutants vs. binding to wild-type enzyme

**Fig. 2:**
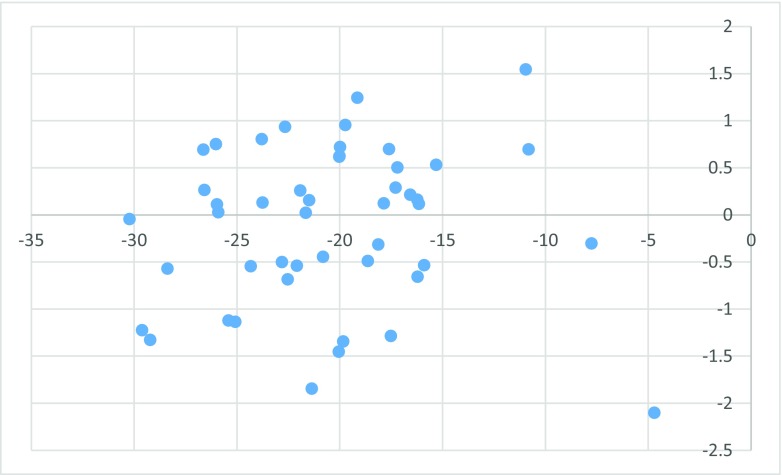
Signed difference in averaged FlexX score between binding to mutants vs. binding to wild-type enzyme as a function of binding score

All 47 ligands present in the PDB that are bound in the allosteric cavity have been docked to all 107 structures and averaged scores for a given ligand were obtained separately for wild-type (wt) and for mutated enzyme (individual data provided in Table S1 in Supporting information). The obtained poses have been inspected for correct orientation within the allosteric cavity (for examples of overlap with the native ligand see Figs. S1 and S2 in the Supporting Information). The results are collected in Table 2. Averaged binding scores have been compared for wt and mutated enzymes. The results are illustrated graphically by Fig. 1. The strong linear correlation obtained indicates that there is no significant difference between binding in either form of the enzyme. Furthermore, as illustrated by Fig. 2, a slight preference for binding in the allosteric pocket of either wt enzyme or its mutated form is random and does not correlate with the energy of binding. The difference is symmetrically distributed between positive and negative values showing practically no systematic preference of binding to either wild-type or one of the mutated forms of the enzyme. Similarly, we have found no correlation between the standard deviation of the average binding score and the binding energy. This observation indicates that activity against mutated HIV-1 RT forms is not governed by the strength of binding. Allosteric ligands impair enzyme action by a wedge mechanism, hindering domain mobility toward opening and closing the access to the active site. However, final allosteric site architecture is achieved upon ligand binding. In order to account for this flexibility and possible clash between the protein and a ligand, we have used large overlap volume (100 Å^3^). Lack of systematic difference between binding to wt and mutated enzyme seems thus to indicate that activity against mutants is connected with the structural features of the ligand rather than their binding energy. Interactions within the allosteric site are mostly associated with van der Waals forces and to a lesser extend to hydrogen bonding [32]. As illustrated by the most suited for mutant enzymes ligand, EFZ, its success seems to come from hydrogen bonding to lysine 101 rather than lysine 103, which is the most frequent mutation (see left panel of Fig. S1).

**Table 2:** Averaged FlexX docking scores for all ligands docked to wild-type (wt) and mutated HIV-1 reverse transcriptase structures

Ligand	Wt	Mutants	Ligand	Wt	Mutants
1BT	−29.6204	−28.397	HBY	−15.3131	−15.8476
5DV	−26.0268	−26.7804	IL5	−22.8162	−22.3174
ETR	−25.9162	−25.9462	IB1	−30.237	−30.1946
AAP	−21.9232	−22.1834	IET	−23.7592	−23.892
AC	−7.76298	−7.46046	JLJ	−25.9829	−26.0958
ADB	−28.3832	−27.8152	KBT	−20.8254	−20.3803
BML	−21.4917	−21.6483	KRL	−26.6452	−27.3403
CXD	−23.7968	−24.6019	KRP	−22.0957	−21.5577
DIZ	−17.6149	−18.316	KRV	−22.5453	−21.8624
EFZ	−10.9564	−12.5018	MRX	−22.6725	−23.6095
EUR	−24.3326	−23.7894	NNB	−20.011	−20.6353
FPT	−21.6605	−21.6845	NNC	−19.9938	−20.7152
FTC	−19.1469	−20.3939	NNI	−20.0292	−20.6486
G73	−29.227	−27.9006	NVE	−4.71138	−2.61201
GFA	−25.0896	−23.9547	NVP	−18.6458	−18.1568
GWB	−17.1987	−17.7041	RPV	−26.5865	−26.8525
GWE	−19.8398	−18.4964	TNK	−20.0555	−18.6039
GWI	−17.5208	−16.2361	TT1	−16.2279	−15.5725
GWJ	−21.369	−19.5249	UCL	−15.9025	−15.3693
H12	−16.2325	−16.396	UDR	−10.828	−11.5263
H16	−16.161	−16.2805	WHU	−25.4155	−24.295
H18	−16.5799	−16.7963	YKN	−18.1383	−17.8257
H20	−17.8664	−17.9892	ZZE	−17.2912	−17.5812
HBQ	−19.7385	−20.696

Based on the results obtained from docking, we have performed QSAR studies. In order to understand the very general structural features affecting the binding affinity, we attempted two approaches to QSAR modeling, aiming for models containing descriptors that have clear “chemical interpretation” and can be easily interpreted to give suggestions for new compound design. In the first approach, we have used “traditional” QSAR that involves a library of descriptors as implemented in the SCIGRESS Suite software.

**Fig. 3:**
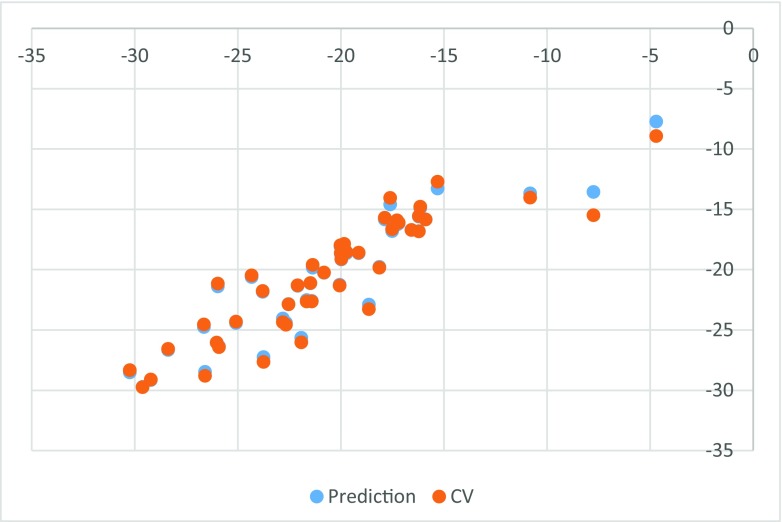
Topological QSAR results obtained with four descriptors

Several models gave acceptable correlation coefficient, while when only easily interpretable descriptors were used the following equation turned out to be statistically the best:

1 $$ {\displaystyle \begin{array}{l} FlexX score=\\ {}-{2.035}^{\ast } double bond count-{3.268}^{\ast}\mathit{\sec}- amine count-{2.570}^{\ast } cyano count+\\ {}6.83817e-{05}^{\ast } nonpolar\ {area}^2-16.375\end{array}} $$

with* r* ^2^ equal to 0.8358; the degrees-of-freedom adjusted* r* ^2^ equal to 0.8202; and the leave-one-out cross-validated* r* ^2^ of 0.7819. The standard deviation in the error predicted by leave-one-out cross-validation is 2.5150. The F-ratio is 53.4646. The probability that a greater F-ratio can be obtained by chance alone is 0.0000. Thus, since the probability is less than 0.05, there is at least one significant descriptor in the model. Partial F values (listed below) indicate relative importance of each descriptor – the larger the value the more important is the descriptor. Topological results are illustrated by Fig. 3.

The ranges of values for the descriptors in the training set and obtained corresponding partial-F values (in parenthesis) were:

- *double bond count* from 4 to 14, (80.156) - SCIGRESS treats aromatic systems as having alternating double and single bonds,
- *sec-amine count* from 0 to 3 (51.719),
- *cyano count* from 0 to 2 (21.880),
- *(nonpolar area)* ^*2*^ from 113,241 to 483,701 Å^2^ (121.969).

Since the objective is to have compounds with the lowest (most negative) FlexX score, the model given by Eq. (1) suggests that molecules should contain nitrile and secondary amine groups, and the area of the molecule incapable of hydrogen bonding (either as a donor or an acceptor) should be as small as possible.

**Fig. 4:**
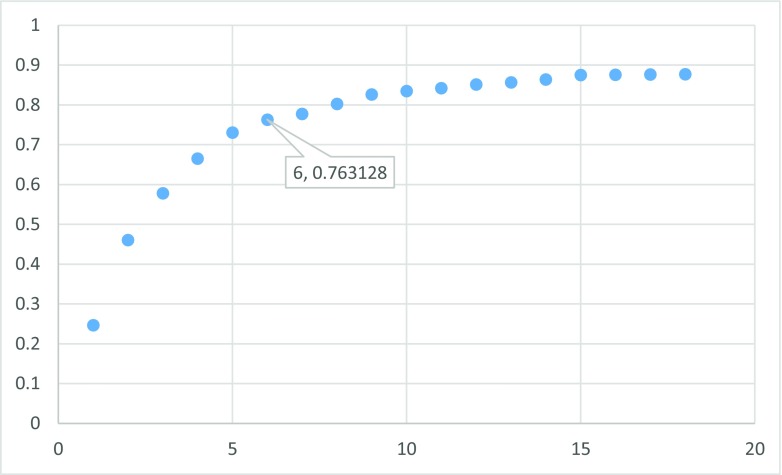
Leaps and bounds graph (*r* ^2^ vs. number of descriptors) illustrating selection of the minimal number of descriptors used for QSAR

**Fig. 5:**
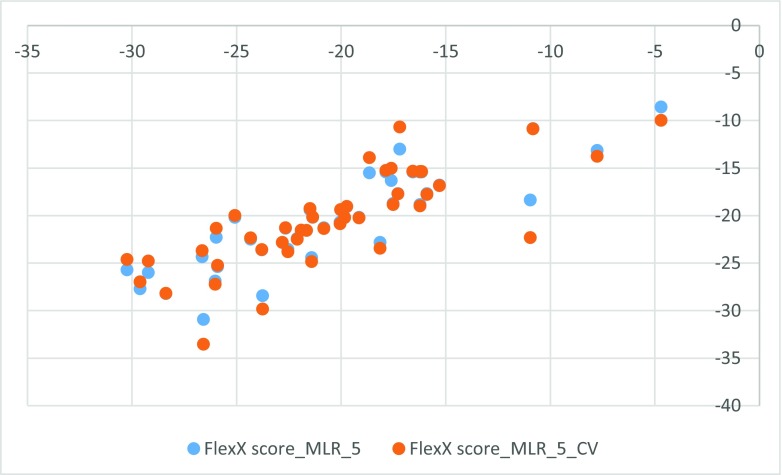
Substructure-based QSAR results obtained with six descriptors

The second attempt aimed at creating QSAR using fragment contribution approach using common substructures present in the training set using ADMEWORKS ModelBuilder. Due to size of the training set, the set of six descriptors was chosen. As illustrated by Fig. 4, this is the lowest number of descriptors that yields acceptable statistically significant results. The set contained X-H (hydrogen attached to any atom) substructure count descriptor. For simpler mechanistic interpretation, the descriptor was manually replaced with C-H count (hydrogens attached to carbon) to calculate the final model. The obtained results are presented in Fig. 5, while the final statistical parameters of this model are collected in Table 3.

**Table 3:** Statistical parameters of the substructure-based model

Basic statistics		Training set	
Number of samples	47	*r* ^2^	76.31
Number of parameters	6	Adjusted* r* ^2^	72.76
Lack of fit (LOF)	4.78	Mean square error (MSE)	7.90
Cross-validation		F-statistic	21.5
n-out	1	*p* value	0.000000
R2CV	60.585	Est. Std Dev. of Mdl Err	2.81

The model cross-validation* r* ^2^ of less than 70% does not encourage its use for direct prediction of unknown compounds. However, the sign of the linear regression equation’s weight vector coefficients is a measure of the influence of a given substructure contribution to activity. In particular, negative values indicate improvement in binding, while positive values suggest that the corresponding substructures should be eliminated or their presence minimized. The obtained results are summarized in Table 4, where substructures with positive contribution to binding are presented in bold, while those which should be avoided are distinguished by italics.

**Table 4:** Results of the substructure-based QSAR model

Structure	Normalized coefficient	Count range in training set
**(33) X -C = C-CN**	−**2.053**	**0–1**
**(30) X2-NCON-X**	−**1.577**	**0–1**
**(11) X-NH-X**	−**1.273**	**0–4**
**(52) X—N-Ph(X)CN**	−**1.092**	**0–1**
*(74)X-Cyclopropyl*	*1.440*	*0–1*
*(1) H-C*	*3.155*	*9–34*

As can be seen, the interpretation of results of the fragment-based QSAR leads to similar conclusions, as the interpretation of the topological QSAR – the presence of sec-amino and cyano groups is improving activity, while parts of purely hydrocarbon-nature should be avoided.

## Conclusions

Two main conclusions are drawn from the research presented in this study:

1. HIV-1 reverse transcriptase (RT) has relatively low fidelity – its error rate is evaluated to be in the range of 10^−5^ per nucleotide addition [33, 34]. A high mutation rate of the virus can result in emergence of strains resistant to antiretroviral drugs. Our docking results indicate that there is no systematic influence of mutations on binding in the allosteric center. Thus, it seems that structural features of the ligand are the main source of the activity toward mutants rather than the binding energy,
2. The interpretation of results of fragment-based QSAR leads to similar conclusions as interpretation of the topological QSAR: the presence of sec-amino and cyano groups is improving binding, while hydrocarbon fragments should be avoided.

## Electronic supplementary material

ESM 1 (XLSX 342 kb)

## References

[1] http://www.unaids.org accessed on 2017–08-08

[2] http://www.who.int accessed on 2017–08-08

[3] Este JA, Cihlar T (2010). Current status and challenges of antiretroviral research and therapy. Antivir Res.

[4] Famiglini V, La Regina G, Coluccia A, Masci D, Brancale A, Badia R, Riveira-Muñoz E, Esté JA, Crespan E, Brambilla A, Maga G, Catalano M, Limatola C, Formica FR, Cirilli R, Novellino E, Silvestri R (2017). Chiral indolylarylsulfone non-nucleoside reverse transcriptase inhibitors as new potent and broad spectrum anti-HIV-1 agents. J Med Chem.

[5] Oguntibeju OO (2012). Quality of life of people living with HIV and AIDS and antiretroviral therapy. HIV AIDS (Auckl).

[6] Zhan P, Pannecouque C, De Clercq E, Liu X (2015). Anti-HIV drug discovery and development: current innovations and future trends. J Med Chem.

[7] Kohlstaedt LA, Wang J, Friedman JM, Rice PA, Steitz TA (1992). Crystal structure at 3.5 Å resolution of HIV-1 reverse transcriptase complexed with an inhibitor. Science.

[8] Arts EJ, Hazuda DJ (2012). HIV-1 antiretroviral drug therapy. Cold Spring Harb Perspect Med.

[9] De Clercq E (2013). The nucleoside reverse transcriptase inhibitors, nonnucleoside reverse transcriptase inhibitors, and protease inhibitors in the treatment of HIV infections (AIDS). Adv Pharmacol.

[10] Ghosh AK, Rao KV, Nyalapatla PR, Osswald HL, Martyr CD, Aoki M, Hayashi H, Agniswamy J, Wang Y-F, Bulut H, Das D, Weber IT, Mitsuya H (2016). Design and development of highly potent HIV-1 protease inhibitors with a crown-like oxotricyclic core as the P2-ligand to combat multidrug-resistant HIV variants. J Med Chem.

[11] Kang D, Fang Z, Huang B, Lu X, Zhang H, Xu H, Huo Z, Zhou Z, Yu Z, Meng Q, Wu G, Ding X, Tian Y, Daelemans D, De Clercq E, Pannecouque C, Zhan P, Liu X (2017). Structure-based optimization of thiophene[3,2-d]pyrimidine derivatives as potent HIV-1 non-nucleoside reverse transcriptase inhibitors with improved potency against resistance-associated variants. J Med Chem.

[12] Chen X, Zhan P, Li D, De Clercq E, Liu X (2011). Recent advances in DAPYs and related analogues as HIV-1 NNRTIs. Curr Med Chem.

[13] Lansdon EB, Brendza KM, Hung M, Wang R, Mukund S, Jin D, Birkus G, Kutty N, Liu X (2010). Crystal structures of HIV-1 reverse transcriptase with etravirine (TMC125) and rilpivirine (TMC278): implications for drug design. J Med Chem.

[14] Janssen PA, Lewi PJ, Arnold E, Daeyaert F, de Jonge M, Heeres J, Koymans L, Vinkers M, Guillemont J, Pasquier E, Kukla M, Ludovici D, Andries K, de Bethune MP, Pauwels R, Das K, Clark AD, Frenkel YV, Hughes SH, Medaer B, De Knaep F, Bohets H, De Clerck F, Lampo A, Williams P, Stoffels P (2005). In search of a novel anti-HIV drug: multidisciplinary coordination in the discovery of 4-[[4-[[4-[(1E)-2-cyanoethenyl]-2,6-dimethylphenyl] amino]-2- pyrimidinyl]amino]benzonitrile (R278474, rilpivirine). J Med Chem.

[15] Ludovici DW, De Corte BL, Kukla MJ, Ye H, Ho CY, Lichtenstein MA, Kavash RW, Andries K, de Bethune MP, Azijn H, Pauwels R, Lewi PJ, Heeres J, Koymans LM, de Jonge MR, Van Aken KJ, Daeyaert FF, Das K, Arnold E, Janssen PA (2001). Evolution of anti-HIV drug candidates. Part 3: Diarylpyrimidine (DAPY) analogues. Bioorg Med Chem Lett.

[16] Cohen CJ, Andrade-Villanueva J, Clotet B, Fourie J, Johnson MA, Ruxrungtham K, Wu H, Zorrilla C, Crauwels H, Rimsky LT, Vanveggel S, Boven K (2011). Rilpivirine versus efavirenz with two background nucleoside or nucleotide reverse transcriptase inhibitors in treatment-naive adults infected with HIV-1 (THRIVE): a phase 3, randomised, non-inferiority trial. Lancet.

[17] Molina JM, Cahn P, Grinsztejn B, Lazzarin A, Mills A, Saag M, Supparatpinyo K, Walmsley S, Crauwels H, Rimsky LT, Vanveggel S, Boven K (2011). Rilpivirine versus efavirenz with tenofovir and emtricitabine in treatment-naive adults infected with HIV-1 (ECHO): a phase 3 randomised double-blind active-controlled trial. Lancet.

[18] Xu HT, Colby-Germinario SP, Asahchop EL, Oliveira M, McCallum M, Schader SM, Han Y, Quan Y, Sarafianos SG, Wainberg MA (2013). Effect of mutations at position E138 in HIV-1 reverse transcriptase and their interactions with the M184I mutation on defining patterns of resistance to nonnucleoside reverse transcriptase inhibitors rilpivirine and etravirine. Antimicrob Agents Chemother.

[19] Xu HT, Asahchop EL, Oliveira M, Quashie PK, Quan Y, Brenner BG, Wainberg MA (2011). Compensation by the E138K mutation in HIV-1 reverse transcriptase for deficits in viral replication capacity and enzyme processivity associated with the M184I/V mutations. J Virol.

[20] Lai MT, Feng M, Falgueyret JP, Tawa P, Witmer M, DiStefano D, Li Y, Burch J, Sachs N, Lu M, Cauchon E, Campeau LC, Grobler J, Yan Y, Ducharme Y, Cote B, AsanteAppiah E, Hazuda DJ, Miller MD (2014). In vitro characterization of MK-1439, a novel HIV-1 nonnucleoside reverse transcriptase inhibitor. Antimicrob Agents Chemother..

[21] Wainberg MA, Zaharatos GJ, Brenner BG (2011). Development of antiretroviral drug resistance. N Engl J Med.

[22] Berman HM, Westbrook J, Feng Z, Gilliland G, Bhat TN, Weissig H, Shindyalov IN, Bourne PE (2000). The Protein Data Bank. Nucleic Acids Res.

[23] Ren J, Bird LE, Chamberlain PP, Steward-Jones GB, Stuart DI, Stammers DK (2002). Structure of HIV-2 reverse transcriptase at 2.35-Å resolution and the mechanism of resistance to non-nucleoside inhibitors. Proc Natl Acad Sci USA.

[24] Stahl M, Rarey M (2001). Detailed analysis of scoring functions for virtual screening. J Med Chem.

[25] Rarey M, Kramer B, Lengauer T, Klebe G (1996). A fast flexible docking method using an incremental construction algorithm. J Mol Biol.

[26] LeadIT 2.1.0, BioSolveIT GmbH, St. Augustin, Germany, 2012

[27] SCIGRESS (2016). Fujitsu Kyushu Systems Limited, Japan

[28] Mercader AG, Duchowicz PR, Fernández FM, Castro EA (2011). Advances in the replacement and enhanced replacement method in QSAR and QSPR theories. J Chem Inf Model.

[29] ADMEWORKS (2014). ModelBuilder.

[30] Mercader AG, Duchowicz PR, Fernández FM, Castro EA (2015). Replacement method and enhanced replacement method versus the genetic algorithm approach for the selection of molecular descriptors in QSPR/QSAR theories. J Chem Inf Model.

[31] Eberhart RC, Kennedy J (1995) A new optimizer using particle swarm theory. Proceedings of the Sixth International Symposium on Micromachine and Human Science, Nagoya, Japan, 39-43

[32] Krzemińska A, Świderek K, Paneth P (2016). Theoretical studies of energetics and binding isotope effects of binding a triazole-based inhibitor to HIV-1 reverse transcriptase. PCCP.

[33] Cuevas JM, Geller R, Garijo R, López-Aldeguer J, Sanjuán R (2015). Extremely high mutation rate of HIV-1 in vivo. PLoS Biol.

[34] Achuthan V, Keith BJ, Bernard A, Connolly BA, DeStefano JJ (2014). Human immunodeficiency virus reverse transcriptase displays dramatically higher fidelity under physiological magnesium conditions in vitro. J Virol.

